# Renal AA-amyloidosis in intravenous drug users – a role for HIV-infection?

**DOI:** 10.1186/1471-2369-13-151

**Published:** 2012-11-21

**Authors:** Oliver Jung, Hans Stefan Haack, Maike Buettner, Christoph Betz, Christoph Stephan, Peter Gruetzmacher, Kerstin Amann, Markus Bickel

**Affiliations:** 1 Department of Nephrology, Goethe University, Frankfurt/Main, Germany; 2 Department of Nephrology, Agaplesion Markus Krankenhaus, Frankfurt/Main, Germany; 3 Institute of Pathology, Nephropathology, Friedrich-Alexander-University, Erlangen, Germany; 4 Department of Infectious Disease, Goethe University, Frankfurt/Main, Germany; 5 Klinikum der Goethe-Universität, Zentrum der Inneren Medizin II, Infektionsambulanz, Haus 68, 1 OG, Theodor Stern Kai 7, 60590, Frankfurt/Main, Germany

**Keywords:** AA-amylodosis, IVDU, Chronic kidney disease, HIV

## Abstract

**Background:**

Chronic renal disease is a serious complication of long-term intravenous drug use (IVDU). Recent reports have postulated a changing pattern of underlying nephropathy over the last decades.

**Methods:**

Retrospective investigation including all patients with prior or present IVDU that underwent renal biopsy because of chronic kidney disease between 01.04.2002 and 31.03.2012 in the city of Frankfurt/Main, Germany.

**Results:**

Twenty four patients with IVDU underwent renal biopsy because of progressive chronic kidney disease or proteinuria. Renal AA-amyloidosis was the predominant cause of renal failure in 50% of patients. Membranoproliferative glomerulonephritis (GN) was the second most common cause found in 21%. Patients with AA-amyloidosis were more likely to be HIV infected (67 vs.17%; p=0.036) and tended to have a higher rate of repeated systemic infections (92 vs. 50%; p=0.069). Patients with AA-amyloidosis presented with progressive renal disease and nephrotic-range proteinuria but most patients had no peripheral edema or systemic hypertension. Development of proteinuria preceded the decline of GFR for approximately 1–2 years.

**Conclusions:**

AA-amyloidosis was the predominant cause of progressive renal disease in the last 10 years in patients with IVDU. The highest rate of AA-amyloidosis observed was seen in HIV infected patients with IVDU. We speculate that chronic HIV-infection as well as the associated immunosuppression might promote development of AA-amyloidosis by increasing frequency and duration of infections acquired by IVDU.

## Background

Renal disease related to intravenous drug use (IVDU) has been reported since the 1970s, mostly in the context of heroin-associated nephropathy, characterized by nephritic syndrome and rapid-progression [1,2]. The incidence of heroin associated-nephropathy decreased in the last four decades. More recent reports starting from the 1990s related chronic kidney disease in IVDU more with concomitant chronic HIV-, HBV- and HCV-infection [3-5]. Two recent studies from Europe observed changing patterns of renal disease in patients with IVDU, reporting an increased prevalence of renal AA-amyloidosis [6,7].

Renal AA-amyloidosis is a complication of chronic and/or recurrent inflammatory disease [8]. Autoimmune diseases, mainly rheumatoid arthritis, are common causes in resource rich countries whereas untreated chronic infections are the predominant cause in countries with limited medical resources. Severe proteinuria, nephrotic syndrome as well as renal insufficiency are the typical clinical manifestations of renal AA-amyloidosis [8]. Successful treatment of the underlying inflammation, by immunosuppressant’s for autoimmune diseases or by antimicrobials for chronic infections, can lead to stabilization of or even improvements of renal function [9].

Although cases of AA-amyloidosis in patients with IVDU have already been described in the 1970s [10,11], only sporadic cases have been reported in the last decades [5,12-14]. To date, it remains unclear to what extent IVDU and associated co-morbidities, especially chronic infections, contribute to the development of renal AA-amyloidosis. Our aim was to describe the histologic pattern of nephropathy and its associated risk factors in a contemporary cohort of patients with IVDU.

## Methods

The Nephrology departments at Goethe-University (GU) and the Agaplesion Markus Krankenhaus (AMK) are two main nephrologic care providers and the only centres conducting renal biopsies in Frankfurt, Germany. All patients with IVDU were identified by using the Patient Data management System of the renal units of the GU and AMK that are based on primary and secondary diagnosis according to the international Classification of Diseases 10th Revision (ICD-10). We then retrospectively analyzed all patients with ongoing or a prior history of IVDU referred to us between 01.04.2002 and 31.03.2012 that underwent renal biopsy because of chronic kidney disease. Biopsies were performed because of progressive renal failure or overt proteinuria. Clinical data and the patient′s medical history were extracted from the medical records. Glomerular filtration rate was estimated (eGFR) using the Modification of Diet in Renal Disease (MDRD) equation [15]. Proteinuria was assessed by collection of 24-h urine at the time of renal biopsy. Proteinuria prior to renal biopsy was determined from spot urine. End-stage renal disease (ESRD) was defined as initiation of chronic renal replacement therapy (RRT) in an ambulatory setting. All data were collected retrospectively; thus no informed consent or approval from the ethics committee was obtained.

Chronic hepatitis B virus (HBV) infection was defined by a detectable HBs antigen persisting at least 6 months. Past HBV exposure was defined by detection of HBV core or surface antibodies in absence of previous vaccination. Chronic hepatitis C virus (HCV) infection was defined by detection of HCV antibodies and positive HCV RNA PCR persisting at least 6 months. HIV infection was defined by positive HIV antibodies or positive HIV RNA PCR.

All renal biopsy specimens were submitted for diagnostic purposes and proceeded using our standardized routine protocol. Briefly, formalin-fixed and paraffin-embedded kidney biopsy specimens were sectioned into 2 μm thick paraffin sections with at least 8 serial sections stained either by periodic acid-Schiff (PAS) or hematoxylin-eosin (HE) stainings. Additionally, a Congo red staining as well as immunohistochemical stainings with antisera specific for IgA (1:150000), IgG (1:100000), IgM (1:75000), C1q (1:75000) and C3c (1:75000, all polyclonal rabbit antisera, Dako Cytomation, Hamburg, Germany) were performed of all cases. In Congo red positive cases additional stainings with antibodies specific for the kappa (1:50000) and lambda (1:100000) light chains (both polyclonal rabbit antisera, Dako) and for amyloid A (1:500, monoclonal, mouse, clone mc-1, Dako) for differentiation of the amyloid deposits were initiated.

Continuous variables were expressed as median and interquartile-range (IQR) or as proportions as appropriate. Continuous and categorical variables were compared for univariate analysis between groups using the *t*-test or Mann–Whitney *U*-test and Fisher exact test, respectively. All p-values reported are two-sided. Statistical significance was assumed when the p-value was <0.05. A statistical trend was assumed when the p-value was >0.05 but <0.08. Kaplan-Meier (Mantel-Cox log-rank test).estimates were used to analyze time to death or progression to ESRD.

## Results

### Patients characteristics and renal findings on biopsy

Between 01.04.2002 and 31.03.2012 a total of 27 IVDU patients were referred to our units because of chronic kidney disease with either progressive renal failure or large proteinuria. Renal biopsy was performed in 24 (88.9%) of these patients. A renal biopsy was not performed in one patient because of impaired coagulation due to liver cirrhosis and two patients declined renal biopsy. Patients were followed after renal biopsy for a cumulative observation time of 59.5 patient-years (range 4 to 3540 days). All patients were Caucasians and mostly male (62.5%). Self reported duration of IVDU was 3–33 years and 12 patients (50%) had been, at least intermittently, on opioid maintenance therapy. Chronic viral infections were frequent: 21 patients (87.5%) had a chronic HCV infection, 10 patients (41.7%) an HIV infection and one (4.2%) had chronic HBV infection. Seven patients (29.2%) had serologic findings indicating prior HBV exposure.

Twelve patients (50%) had renal AA-amyloidosis and five patients (20.8%) had a membranoproliferative glomerulonephritis type I. All other types of renal disease were seen only in 1 or 2 patients (Table 1).

**Table 1:** Patients characteristics and renal findings on biopsy

Age (years)	41 (33 – 46)
Male – n (%)	15 (62.5%)
Self-reported time after initiation of IVDU (years)	22 (13 – 30)
Self-reported duration of IVDU in years	17 (8 – 25)
History of opioid maintenance therapy	12 (50%)
Chronic hepatitis B	1 (4.2%)
Past HBV exposure	7 (29.2%)
Chronic hepatitis C	21 (87.5%)
HIV infection	10 (41.7%)
Renal disease by biopsy-findings – n (%)	
AA-amyloidosis	12 (50.0%)
Membranoproliferative GN Type I	5 (20.8)
Nephrosclerosis	2 (8.3%)
Extracapillary proliferative GN	2 (8.3%)
Focal segmental Glomerulosclerosis	1 (4.2%)
Chronic tubulointerstitial nephritis	1 (4.2%)
Lupus nephritis Class IV	1 (4.2%)

Variables are expressed as median and interquartile-range (IQR) or as proportions as appropriate.

### Clinical characteristics of patients with renal AA-amyloidosis

**Figure 1:**
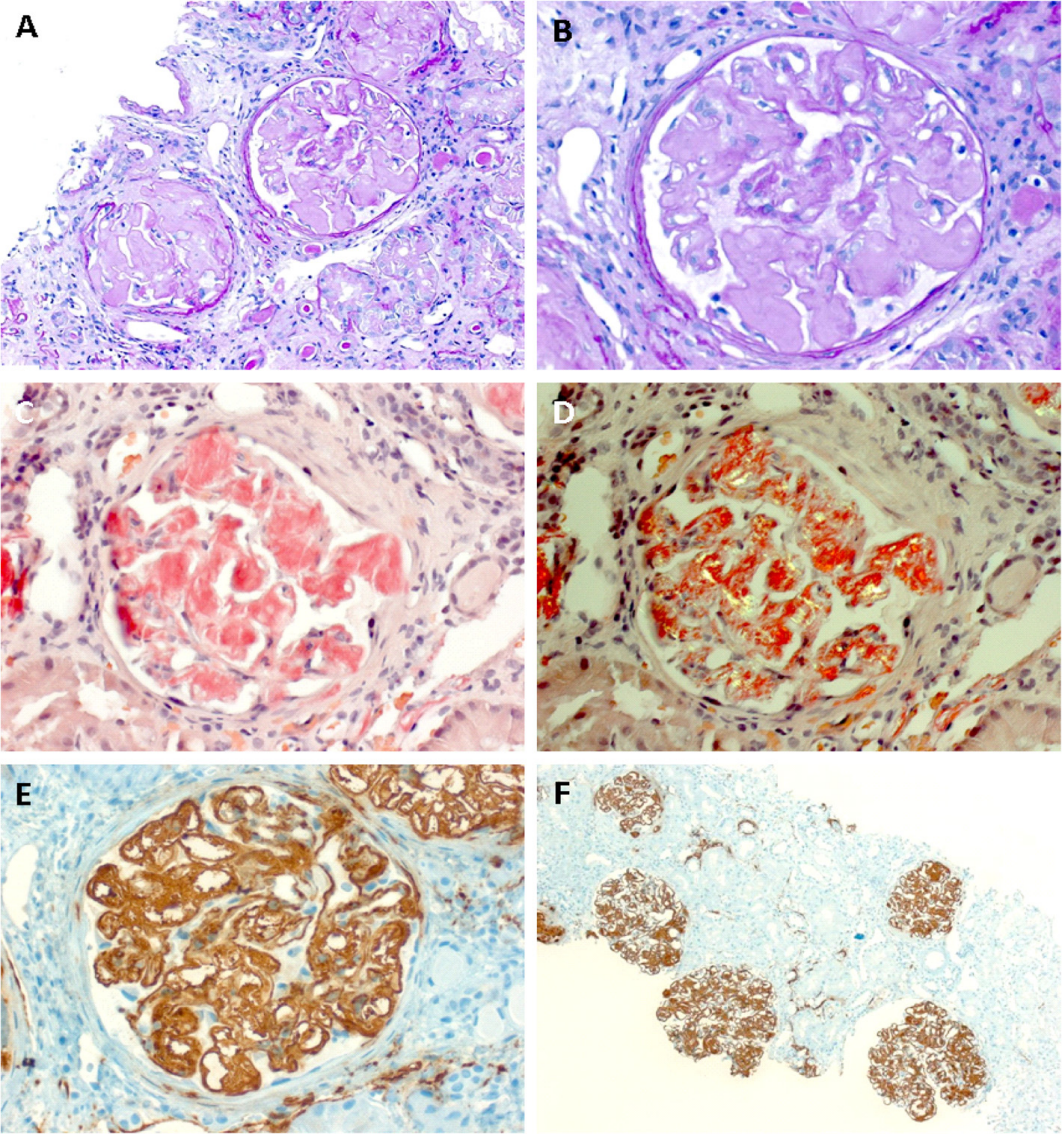
Representative histological findings in a case of renal AA-amyloidosis.

All 12 patients with renal AA-amyloidosis presented with elevated serum creatinine and nephrotic-range proteinuria, but only 3 (25%) had signs of peripheral edema in physical examination. Hypertension was uncommon and found only in one patient (8.3%). Ultrasound examination revealed enlarged kidneys with increased echogenicity in all patients. All patients had a medical history of chronic or repeated infections. Examination of renal biopsies showed deposition of AA-amyloid in the glomerula, the vessels and the interstitial compartment. Tubular atrophy and interstitial fibrosis was present in all patients ranging from 20 to 80% (representative histological findings are presented in Figure 1). Two patients had additional signs of acute interstitial nephritis.

### Time course of renal AA-amyloidosis

**Figure 2:**
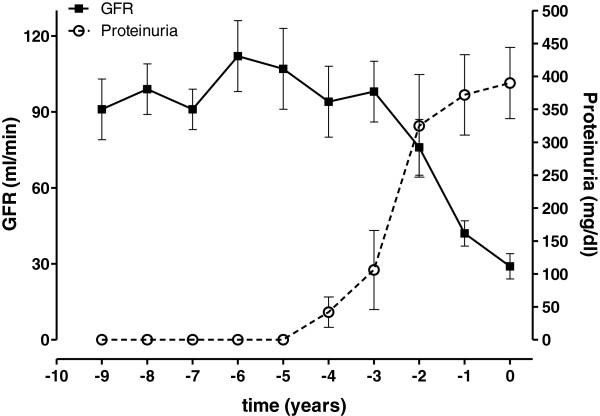
Clinical course of renal AA-amyloidosis prior to renal biopsy.

For 10 out of 12 patients with AA-amyloidosis we were able to retrieve medical records on kidney function prior to the time of renal biopsy. All patients showed a transient period of hyperfiltration followed by a rapid decline of the eGFR within less than 2 years. Proteinuria preceded this eGFR decline a median of 20 months (range 13–36 months). Clinical course of renal AA-amyloidosis prior to biopsy is demonstrated in Figure 2.

**Figure 3:**
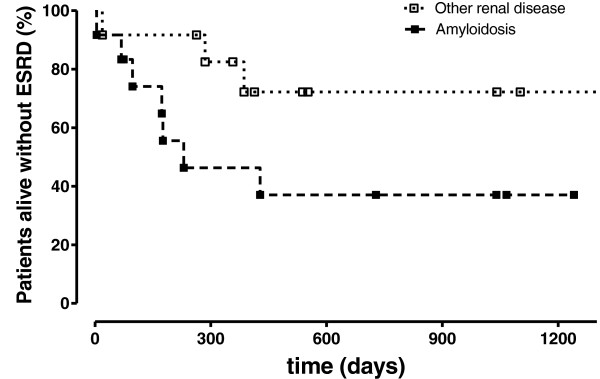
Outcome in patients with diagnosed renal AA-amyloidosis compared to those with other types of disease.

Two patients died within 1 year after diagnosis (one because of sepsis, one of acute drug intoxication), and five additional patients progressed to ESRD within less than 2 years. Among those five patients with ESRD, three patients died within 2 years after initiation of renal replacement therapy (one because of sepsis, one of drug intoxication, one by suicide). Among patients with other causes of renal disease, 3 patients progressed to ESRD. Outcome of patients with established diagnosis of renal AA-amyloidosis is shown in Figure 3, demonstrating the poorer outcome of these patients (p=0.0782).

Four surviving patients did not progress to ESRD within more than 2 years after the diagnosis; all had stopped IVDU after diagnosis of AA-amyloidosis and were successfully treated with opioid maintenance therapy.

### Comparison of patients characteristics between those with or without renal AA-amyloidosis

Both groups of patients were comparable for most demographic and clinical characteristics (Table 2). Amount of proteinuria was higher in patients with renal AA-amyloidosis as compared to those with other types of renal disease (p=0.021). HIV infection was significantly more frequent in patients with AA-amyloidosis than in those with other types of chronic renal disease (67 vs. 17%; p=0.036). Patients with AA-amyloidosis tended to have a longer self-reported duration of IVDU (20.5 vs. 12.5 months; p=0.056) and a higher frequency of chronic or repeated infections (92 vs 50%; p=0.069) (Table 2). Of the eight patients with HIV infection and renal AA-amyloidosis, three patients had previous AIDS-defining events and four patients were previously naïve to antiretroviral therapy at the time of renal biopsy (in two the HIV infection was first diagnosed at the time of renal biopsy).

**Table 2:** Comparison of patient’s characteristics

	**AA-Amyloidosis**	**Other renal**	**p-value**
	**(n=12)**	**disease (n=12)**	
Age (years)	39.5 (32 – 44)	43.5 (33 – 48)	0.563
Male – n (%)	9 (75.0%)	6 (50%)	0.400
Self-reported time after initiation of IVDU (years)	22 (17 – 31)	19.5 (11 – 29)	0.418
Self-reported duration of IVDU (years)	20.5 (14 – 27)	12.5 (7 – 19)	0.056
History of opioid maintenance therapy	5 (41.7%)	7 (58.3%)	0.684
Chronic hepatitis B	1 (4.2%)	0 (0%)	1.0
Chronic hepatitis C	10 (83.3%)	11 (91.7%)	1.0
HIV	8 (66.7%)	2 (16.7%)	**0.036**
Medical history of			
Chronic or repeated skin infections	11 (91.7%)	6 (50.0%)	0.069
Bacterial endocarditis	2 (16.7%)	1 (8.3%)	1.000
Repeated pneumonia	8 (66.7%)	3 (25.0%)	0.100
Serum creatinine (mg/dl)	2.5 (1.9 – 4.0)	2.4 (1.7 – 3.2)	0.902
Proteinuria (g/24 h)	8.9 (7.5 – 19.1)	4.1 (1.5 – 9.8)	**0.021**
Serum albumin	2.2 (1.5 – 2.8)	3.0 (2.0 – 3.9)	0.124
Total serum protein	6.2 (5.3 – 7.2)	6.1 (4.9- 7.4)	0.853
Peripheral edema	3 (25.0%)	4 (33.3%)	1.0
Hypertension	1 (8.3%)	5 (60.0%)	0.1

Variables are expressed as median and interquartile-range (IQR) or as proportions as appropriate.

Because of the significant relationship between HIV and renal AA-amyloidosis in this cohort we compared these data to those of 25 HIV-positive patients without a prior history of IVDU that underwent renal biopsy within the same time range, and found none of them having renal AA-amyloidosis (data not shown).

## Discussion

In this retrospective study we found renal AA-amyloidosis to be the predominant cause of progressive kidney disease in IVDU accounting for 50% of cases, followed by membranoproliferative glomerulonephritis as the second leading cause in 20.8%. This finding is well in accordance with two recent observations from other European Centres. These studies described an increasing rate of renal AA-amyloidosis in IVDU, while in older biopsy studies AA-amyloidosis was found only sporadically, indicating a changing pattern of nephropathy in this patient population [5-7].

The occurrence of renal AA-amyloidosis in IVDU has been linked to repeated bacterial infections, especially to skin and soft tissue infections, endocarditis as well as longer survival time after the onset of IVDU (as a consequence of successful opoid maintenance therapy) [6,7,10,11]. When comparing patients with renal AA-amyloidosis to those without, we found a trend towards a longer self-reported time of IVDU. Repeated infections tended also to be more frequent in patients with AA-amyloidosis. Yet, the low number of patients in our series makes it difficult to establish a link between isolated sites of infection and occurrence of AA-amyloidosis. Alternatively, it might be the case that not one specific focus but the mere total number of bacterial infections predisposes to the development of renal amyloidosis.

The proportion of patients with HIV infection was significantly higher in patients with renal AA-amyloidosis. This observation has not been made in two published studies on AA-amyloidosis in patients with IVDU. One study did not provide data concerning HIV serostatus, while the other found only one out of 20 patients to be HIV infected [6,7]. However, this does not rule out a higher rate of HIV infections in these studies, as in some of our patients the HIV infection was just diagnosed before the renal biopsy was conducted. Several cases of amyloidosis in HIV infected IVDU have been described in the literature [5,6,13,14,16,17]. Besides IVDU, renal amyloidosis in HIV infected patients has been described in association with visceral leishmaniosis or multicentric Castleman`s disease [18-21], which were both not found in our cohort. During the same time period of this retrospective study, 25 HIV-infected patients without a history of IVDU underwent renal biopsy at our institutions and none of them had evidence of renal AA-amyloidosis [data not shown]. Although these patients cannot be properly matched, because of a higher median age and 25% being of African-descent, this could indicate that the HIV infection by itself does not predispose for AA-amyloidosis, but coeval IVDU might increase the risk. Indeed, in a recent study, we found IVDU to be an independent risk factor for ESRD in an HIV-infected population [22]. Moreover, another study demonstrated that the combination of HIV infection and IVDU increases the risk for chronic kidney disease (CKD) in IVDU, with a higher prevalence of proteinuria in HIV-infected compared to uninfected IVDU [23]. The majority of HIV infected patients with renal AA-amyloidosis had a poorly or uncontrolled HIV infection prompting a severe immunosuppression and a chronic inflammation. Thus the uncontrolled HIV infection might be an important direct and indirect factor for the development of AA-amyloidosis predisposing to more frequent and severe bacterial infections.

Although there are several case reports of AA-amyloidosis in HIV infected IVDU, such a high rate has not been previously described. What might explain this finding? First, medical management of the IVDU population is challenging, as patients often do not return for follow-up and seek medical advice only when acutely ill. Hence, renal biopsy is infrequently performed in IVDU. Second, the time course of renal amyloidosis, its clinical presentation with lack of peripheral edema or systemic hypertension, and enlarged echogenic kidneys on ultrasound makes it indistinguishable from certain other types of renal disease, especially HIV-associated nephropathy (HIVAN) [3,17,24]. The diagnosis of HIVAN is often established solely clinically and is often not confirmed by renal biopsy. Thus the true prevalence of renal AA-amyloidosis might even be underestimated [25,26]. Third, development of AA-amyloidosis also depends on predisposing genetic factors and is more frequent in Caucasian compared to Black patients. Vice versa HIVAN occurs mostly in Black patients and is a rarity in Caucasians [27]. Thus different ethnic compositions of IVDU cohorts may also contribute to the higher incidence observed by us and other European centres when compared to studies from the US with far higher rates of black IVDU [6,7].

IVDU patients with AA-amyloidosis had a poor prognosis, when compared to patients with other underlying causes of AA-amyloidosis [6-8]. We and others found that patients, who remained abstinent from IVDU after the initial diagnosis, achieved stable or even improving renal function during follow-up. This supports the idea that removal of the recurrent inflammatory stimuli can lead to at least some reversal of disease [6]. These observations, underscore the importance of renal biopsies, especially in the light of the oligosymptomatic clinical presentation of these patients. We found that overt proteinuria preceded the GFR decline by approximately 1–2 years. But unfortunately urine analysis is often missed by IVDU because unannounced illicit drug testing with possible consequences for opioid maintenance therapy is feared.

Besides the already discussed, our study has several limitations. First, the population studied is small and from a single metropolitan area. Thus, our results cannot be generalized to other HIV, often ethnically highly divers, cohorts. Second, IVDU often consume more than one substance of varying, uncontrolled purity. Thus our retrospective study cannot determine possible differences between substances used.

## Conclusion

In conclusion we found AA-amyloidosis was the predominant cause of progressive renal disease in the last 10 years in IVDU patients. The high incidence of AA-amyloidosis observed was related to coeval HIV infection. We speculate that the HIV mediated immunosuppression promotes development of AA-amyloidosis by increasing frequency and severity of infectious complications acquired by ongoing IVDU. Overt proteinuria preceded the decline of GFR for approximately 1–2 years, providing an opportunity for early intervention in an otherwise fatal disease.

## Competing interests

All authors declare that they have no competing interests and have had no involvements that might raise the question of bias in the work reported or in the conclusions, implications, or opinions stated. No funding was obtained for this study.

## Authors’ contributions

OJ and MB initiated the study, treated patients, collected and analyzed the data and wrote the manuscript. MBüttner and KA examined the renal biopsies. All other authors treated the patients and are substantially involved in the collection and monitoring of data. No funding was obtained for this study. All authors read and approved the final manuscript.

## Pre-publication history

The pre-publication history for this paper can be accessed here:

http://www.biomedcentral.com/1471-2369/13/151/prepub
